# Enabling Health Information Recommendation Using Crowdsourced Refinement in Web-Based Health Information Applications: User-Centered Design Approach and EndoZone Informatics Case Study

**DOI:** 10.2196/52027

**Published:** 2024-05-29

**Authors:** Wenhao Li, Rebecca O'Hara, M Louise Hull, Helen Slater, Diksha Sirohi, Melissa A Parker, Niranjan Bidargaddi

**Affiliations:** 1 College of Medicine and Public Health Flinders University Clovelly Park Australia; 2 School of Information Science and Engineering Shandong Normal University Jinan China; 3 Robinson Research Institute Adelaide Medical School University of Adelaide Adelaide Australia; 4 Curtin School of Allied Health Curtin University Perth Australia; 5 Canberra Endometriosis Centre Centenary Hospital for Women and Children Canberra Australia

**Keywords:** information recommendation, crowdsourcing, health informatics, digital health, endometriosis

## Abstract

**Background:**

In the digital age, search engines and social media platforms are primary sources for health information, yet their commercial interests–focused algorithms often prioritize irrelevant content. Web-based health applications by reputable sources offer a solution to circumvent these biased algorithms. Despite this advantage, there remains a significant gap in research on the effective integration of content-ranking algorithms within these specialized health applications to ensure the delivery of personalized and relevant health information.

**Objective:**

This study introduces a generic methodology designed to facilitate the development and implementation of health information recommendation features within web-based health applications.

**Methods:**

We detail our proposed methodology, covering conceptual foundation and practical considerations through the stages of design, development, operation, review, and optimization in the software development life cycle. Using a case study, we demonstrate the practical application of the proposed methodology through the implementation of recommendation functionalities in the EndoZone platform, a platform dedicated to providing targeted health information on endometriosis.

**Results:**

Application of the proposed methodology in the EndoZone platform led to the creation of a tailored health information recommendation system known as EndoZone Informatics. Feedback from EndoZone stakeholders as well as insights from the implementation process validate the methodology’s utility in enabling advanced recommendation features in health information applications. Preliminary assessments indicate that the system successfully delivers personalized content, adeptly incorporates user feedback, and exhibits considerable flexibility in adjusting its recommendation logic. While certain project-specific design flaws were not caught in the initial stages, these issues were subsequently identified and rectified in the review and optimization stages.

**Conclusions:**

We propose a generic methodology to guide the design and implementation of health information recommendation functionality within web-based health information applications. By harnessing user characteristics and feedback for content ranking, this methodology enables the creation of personalized recommendations that align with individual user needs within trusted health applications. The successful application of our methodology in the development of EndoZone Informatics marks a significant progress toward personalized health information delivery at scale, tailored to the specific needs of users.

## Introduction

### Background

Members of the general public predominantly resort to search engines such as Google or social media platforms such as Facebook, YouTube, and TikTok as their initial source of health information [1-7]. These platforms use intricate recommendation algorithms to curate the information made accessible to users [8]. The algorithms are designed to rank information based on certain criteria, presenting it in the order of the ranking score. However, the underlying architecture of these ranking systems is by default crafted with commercial intent as opposed to health-centered intent. As a result, information that entices interactions that lead to increased revenue, such as more time spent on the platform or increased traffic to advertisements, gets ranked more prominently. Meanwhile, the information that accurately reflects people’s medical needs is buried under large amounts of unrelated articles and posts and becomes difficult to find [9,10].

As the preference for web-based information seeking continues to grow, the development of web-based health information applications by trusted sources has become increasingly popular [11,12]. Through these applications (eg, websites or mobile apps) [13], people can readily access a wealth of health information generated by trusted sources. These interactions present an opportunity to shape an alternative ranking architecture for recommending web-based health content, one that is grounded in health outcomes. The information curated by these trusted platforms is considered superior in quality. Using user behavior after content access to rank health information could pave the way for more effective algorithms. This improved method could be integrated into search engine and social media algorithms through regulatory measures, challenging the current prioritization of web-based health content.

The existing body of research lacks comprehensive guidance on integrating content-ranking algorithms into applications centered around health information delivery. In this paper, we outline a generic methodology to guide the design and implementation of health information recommendation functionality within web-based health information applications. In this methodology, the health information recommendation interface and logic are co-designed with medical experts and application users such as patients and their supporters. This ensures the credibility of the health information provided, as well as accurate reflection of users’ preference when interacting with the application. The health information recommended to users is ranked and presented using crowdsourcing technology based on feedback from users who have similar demographic and medical profiles. This ensures that health information can be delivered to people according to their situations and needs. The methodology can be easily integrated into new or existing health information applications. By implementing this ranked health information recommendation feature, we foresee improvements in user experience (UX) and the relevance of health information provided.

This methodology for enabling health information recommendation was first formulated based on our experience and expertise in informatics system development and implementation. It was then further refined and validated through the process of designing and implementing the informatics features of a medical information platform named EndoZone [14]. The platform is funded by the Australian government and Jean Hailes for Women’s Health and provides evidence-based information to address symptoms and strategies for managing endometriosis. We illustrate the applicability of the methodology through its application in the EndoZone platform to enable its tailored health information recommendation system known as EndoZone Informatics. The implementation process shows that the methodology is practical for enabling information recommendation functionalities for web-based health information applications that have targeted health content–sharing requirements. Early data show that the solution built using this methodology is effective in reflecting users’ feedback and providing highly personalized information recommendations and is also highly flexible in adjusting information recommendation logic. It has also been observed that the design of the user engagement process and user interface (UI) is highly relevant to the rate of users providing feedback and hence can affect the outcome of an information recommendation solution significantly.

The aim of this paper was to outline a generic methodology to guide the design and implementation of health information recommendation functionality within web-based health information applications and demonstrate its application in designing and implementing the informatics features of the EndoZone health information platform.

### Related Research

A substantial amount of the articles and videos recommended by search engines and social media platforms have quality issues. They may contain biased content, are not comprehensive enough to cover the topic, are not evidence based, and provide limited coverage or content irrelevant to the topic [5,6,9,10,15]. A review by Osman et al [6] highlighted that >40% of the videos on YouTube on lumbar discectomy, cardiopulmonary resuscitation, and stroke are not useful, while more than half of the videos about vaccination as well as phototherapy and excimer laser treatment for psoriasis reflect bias due to commercial interests. A study assessing the quality of diabetes-related content on TikTok found that the quality of the content varies significantly depending on the types of creators and does not fully meet the health information needs of patients [5]. From billions of web pages and videos on the internet, commercial recommendation algorithms of search engines and social media platforms show those with the highest rank first, where the ranking criteria often have nothing to do with whether the content could meet people’s medical needs [16]. To obtain a higher rank, which leads to a higher visibility rate and eventually a better commercial outcome, billions of dollars have been invested by companies for search engine optimization [8]. This compounds the situation because trusted health information sources such as research organizations and noncommercial health organizations often do not have the financial capacity to compete with commercial companies. As a result, the recommendations made by search engines and social media platforms lead people to unrelated articles, commercial advertisements, or even misinformation. As people generally lack the skills and experience to evaluate the accuracy of the information they are recommended [17], incorrect and harmful medical decisions could be made.

In comparison, web-based health information applications developed by trusted sources such as governments, credited health organizations, and universities provide health information with criteria that people value, such as trustworthiness, expertise, and objectivity [18]. In recent years, many of these applications have been developed globally to bypass the information recommendation algorithms of search engines and social media platforms [11,12,19,20]. Several applications contain mechanisms that provide personalized recommendations of nutritional information, medications, treatment plans, diagnoses or disease predictions, physical activities, or other health care services, based on users’ profiles and inputs [21]. However, these recommendation features have not yet been applied extensively in health informatics and medical scenarios [22] and are typically created on an app-to-app basis, targeting a specific disease or recommendation context [12].

The lack of effective information recommendation functionality can be eliminated by enabling health information recommendation capability at scale. Many web-based health information applications could apply similar methodologies in design, development, and evaluation in terms of health information recommendation functionality due to their similarities in context, purpose, and category of recommended items. Tran et al [12] summarized 4 basic recommendation techniques: collaborative filtering, content-based filtering, knowledge-based recommendation, and a hybrid recommendation that combines these 3 techniques. In terms of evaluating the recommendation quality and the effectiveness of the recommendation mechanism, users’ feedback is considered to be a major quality criterion [23]. Crowdsourcing technology has been applied in health care and has proven to be an effective approach to collecting retrospective data, such as user feedback, from a large number of dispersed participants [24]. With the development of health informatics technology and current trends of population preferences toward seeking information on the web, the use of crowdsourcing technologies for validating the effectiveness of health information recommendations is promising.

## Methods

### Overview

In this section, we present the concept of the methodology as well as implementation-related design, including software components, software development, and maintenance considerations, during 2 different implementation phases.

**Figure 1:**
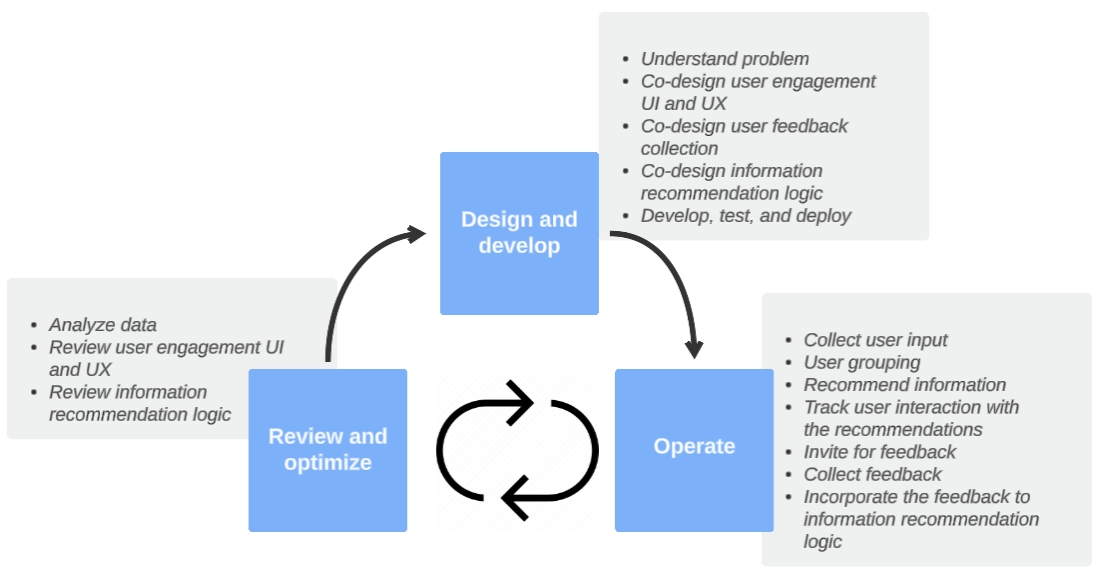
The information recommendation solution life cycle. UI: user interface; UX: user experience.

The methodology for enabling health information recommendation functionalities involves medical experts, researchers or data analysts, software developers, designers, and users of the web-based health information application. As shown in Figure 1, the methodology consists of 3 stages: *design and develop*, *operate*, and *review and* *optimize*. At a high level, the methodology can be summarized thus: first, co-design and codevelop the information recommendation solution; second, recommend information to, and collect feedback from, users to improve the recommendation logic; and third and last, periodically review the statistical data to identify issues and continually adjust the solution.

### Design and Develop

#### Overview

**Figure 2:**
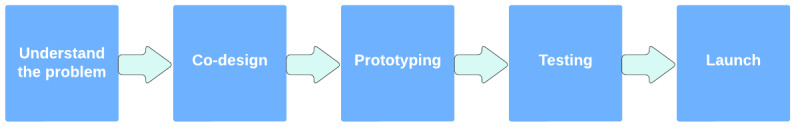
The design and development process.

The implementation of the health information recommendation functionalities in the web-based health information application starts with the design and development of a solution that meets the specific requirements of the application. The design and development process adopts a human-centered design thinking model [25,26]. It considers the need of users to be the main factor that drives the design decision-making process. Figure 2 shows the design and development process for the methodology.

The solution designer first needs to understand the problem and develop detailed requirements for the information recommendation functionalities. To engage in the cocreation process, users can be invited to participate in observation sessions or interview sessions to explore the key challenges and their needs. During these sessions, the questions to be answered could include *What problem is the application trying to solve? What is the status of the application (launched/unbuilt)? What information will be recommended? How is the information expected to be recommended? What data are available for the recommendation logic to be based on?* and *If the application already exists, what does it look like and how is the information recommendation component expected to be integrated?*

Once all requirements have been clearly defined, a series of co-design or ideate sessions are carried out by the solution designer and the medical experts who have comprehensive domain knowledge about the condition or disease the application covers. The co-design process aims to deliver several outcomes, as outlined in the following subsections.

#### The Engagement Process

This is also called UI or UX, that is, the UI and the process that the user interoperates with the application’s information recommendation functionalities. It will need to be co-designed with medical experts to fully consider users’ medical needs.

#### The Feedback Collection Method

This includes the feedback questions to be asked and the form of questions to be delivered, and it needs to be co-designed with the medical experts as well as the researchers or data analysts, making sure that good UXs and the data collected can properly serve the purpose of the feedback collection.

#### The Information Recommendation Logic

This specifies how health information that is recommended to users can be realized via different data structures and algorithms. On the basis of the EndoZone Informatics example that we will present later, the recommendation logic could include things such as a list of expert-verified information, a set of rules for information recommendation, an algorithm for user grouping, an algorithm for feedback analysis, an algorithm for feedback incorporation, and an algorithm for information recommendation. In the co-design process for this deliverable, medical experts should be closely involved in the design of all included components, providing insights that are as detailed as possible and making sure that the recommendations are appropriate (ie, evidence based) and meet users’ medical needs. Specifically, the list of expert-verified information and the set of rules for information recommendation should be based on medical experts’ input and available research data. Taking a rule in EndoZone as an example, a recommendation of yoga as a self-management strategy is made for a user who has severe pelvic pain and does not experience heavy bleeding during menstruation. In addition to medical experts, researchers or data analysts should also participate in the co-design process, making sure that the algorithms are correctly designed.

#### The Develop, Test, and Deploy Processes

To ensure that the design fully reflects the users’ needs while fully considering the complexity and professionalism of the design activities, a smaller group of user representatives can be invited for consultation, where staged co-design outcomes, as mentioned previously, are sent for review and feedback.

After the co-design process is completed, the solution designer translates the outcomes into system design and architecture specifications, which are then used by software developers to develop, test, and deploy the system. How the develop, test, and deploy processes are carried out depends on the preference of the software development team, where no restrictions are imposed by the methodology. However, it is necessary for stakeholders, including researchers, medical experts, and users, to participate in testing early versions of the solution and provide feedback, where design issues and recommendation logic issues can be identified and resolved in time. The tests can be carried out differently by different stakeholder groups; for example, medical experts and researchers can be asked to test specific features that are closely related to their expertise, while for users of the application, a series of tasks that match their needs and expectations (provided in the initial requirement collection or understanding sessions) can be preset, making sure that their feedback is relevant and targeted.

This *design and develop* stage may be conducted multiple times throughout the lifespan of an information recommendation solution in which the solution is updated to fix issues that are identified and rectified or to incorporate new features.

### Operate

#### Overview

The methodology shifts to the *operate* stage once the information recommendation functionalities are launched. In this stage, the solution performs activities such as recommending health information to users, collecting user feedback on recommendations, and incorporating the feedback into the recommendation logic. The first entry point of the users to the solution should be an event that is related to the content of the application; for example, it can be a click on a button on a web page, an action when using a digital tool, or a click on a link included in an invitation email. Such an event triggers a series of activities to generate a list of recommendations to the user, as outlined in the following subsections.

#### Collect User Data on Entry

Data stored in the application, such as user account profiles, user input in digital tools, and user browsing history, contain information about the unique circumstance of a user that is needed for personalizing recommendations. When the entry-point event happens, such data are collected for subsequent algorithms.

#### Group Users

This is an essential step for recommending personalized information to users with different conditions. In this step, the users are grouped by the algorithm for user grouping, based on a set of predefined attributes. Members of a group could have similar demographic and medical profiles, such as condition, age, educational background, symptoms, treatments, and so on.

#### Recommend Information

Using the data collected on entry as input, an algorithm generates a list of recommended information according to the information recommendation logic. In the algorithm, first, user data collected on entry are checked against the rules for information recommendation; for example, if a user *U* has symptom *S*, and the rule *R* indicates that all users with symptom *S* will be recommended information *I*, then *I* will be recommended to user *U*. Second, recommendations that match the rules will be ranked according to previous feedback from all users in the same user group. Third and last, the information is shown to the user in the order of the rank, where information with the best feedback (eg, the highest positive feedback rate) is presented first and has a better chance to be viewed.

#### Track User Interaction

After the information has been recommended, users are likely to read not all but a subset of the recommendations. It is necessary to track which recommendations are read by a user so that in the later step of collecting user feedback to recommendations, questions can be asked effectively. It is assumed that a recommendation has been read by the user if the content has been exposed to the user (eg, the user clicks on a link to an article). Therefore, any interaction that indicates exposure of the information to the user is recorded. Depending on the UI or UX design, recorded interactions could include clicks on a recommendation link or button, the opening of the web page of the recommendation, and so on.

The evaluation of whether the recommendations meet users’ medical needs relies on feedback from the users themselves, supported by the power of crowdsourcing. After a certain period of making the recommendations, attempts are made to collect summative feedback from users who may have read the recommendations and potentially carried out practical activities based on the recommendations.

#### Invite for Feedback

The collection of feedback starts with sending an invitation to the user for participation. If the user has accessed any of the recommendations, an invitation for feedback is sent. Invitations can be sent in the form of oral invitations (eg, telephone invitations or opportunistic face-to-face invitations) or written letter invitations (by post or via email), which will vary from case to case [27]. The method of sending invitations is determined according to medical experts’ suggestions to approach users with specific medical conditions appropriately and maximize the response rate.

#### Collect Feedback

There are a few ways in which user feedback data can be collected on the web (eg, conducting web-based surveys and allowing user ratings) [28]. Conducting a web-based survey is one of the most popular ways to collect user feedback, is easy to implement, and can meet the requirements of a web-based health information application in many cases. Questions in the survey can be asked from a UX perspective in terms of the helpfulness of the recommendations; for example, questions against a therapy recommendation could include *Did you try this therapy? Did you find the therapy easy to do? How difficult did you find fitting this therapy into your life with your other activities?* and *Did you find this therapy helped in managing your symptoms?* One of the known issues of web-based surveys is the low completion rate [29]. Some strategies to incentivize completion rates can be found in existing studies [30,31].

#### Incorporate

After a user’s feedback is collected and digitized, first, an algorithm for analyzing feedback executes to convert the feedback data into measurable attributes. Second, an algorithm for feedback incorporation deploys these attributes into the information recommendation logic. Depending on the design of the algorithm for feedback incorporation, the outcomes of the incorporation could include an updated set of rules for information recommendation, updated ranks of recommendations, updated descriptions for each recommendation, and so on. After the incorporation process finishes, the user’s journey with the information recommendation solution is completed. The updated information recommendation logic will then be applied when other users engage with the information recommendation solution.

### Review and Optimize

#### Overview

As the information recommendation solution operates, system operation data and user engagement data accumulate. Besides using the user interaction data for improving the information recommendation logic in the *operate* stage, an in-depth review and optimization of the solution can be conducted. The purpose is to identify issues based on the analytical outcome of the accumulated operation data set and the experience gained from the continuous operation and maintenance of the solution. Whether the *review and* *optimize* stage needs to be carried out depends on several factors, such as the amount of analyzable data accumulated, the urgency of major optimization of the solution to address emerging requirements, and the operation status of the current information recommendation solution. Researchers and software developers need to decide when a formal *review and* *optimize* stage is needed. The outcome of the *review and* *optimize* stage should include an optimization plan, where detailed redesign and development can be carried out in the following *design and develop* stage.

#### Analyze Data

User engagement data such as user profile data, data of user interaction with the information recommendation solution, and user feedback as well as system operation data such as operation logs and web-based traffic data are accumulated and of statistical value to the optimization of the information recommendation solution. Depending on the sufficiency of the accumulated data, research questions such as *Do the users engage well with the solution? Is the recommendation solution effective in helping users to find the information that meet their medical needs?* and *Are the recommendations appropriate and suitable for the user to practice?* can be answered, and potential issues in the engagement process and recommendation content can be identified.

#### Review User Engagement UI and UX

At the *review and* *optimize* stage, a retrospective review can be conducted toward the user engagement UI and UX. The review can be based on 2 sources of input: first, it can be based on the researchers’ experience gained while continuously operating the user engagement UI and UX. Second, it can be based on user feedback, such as volunteer user group feedback when asked to test and promote the solution. This review could identify design issues in the UI and user engagement process that cause difficulty for users in accessing the features of the solution and the health information they are recommended.

#### Review Information Recommendation Logic

It is difficult to provide the best configuration to elements of the information recommendation logic and achieve the optimal recommendation outcome during the *design and develop* stage. The reasons include users’ composition, uncertainty in user interaction patterns with the application, and a lack of analyzable data. Thus, continuous adjustments to the configuration of algorithms and data structures are needed; for example, grouping attributes and the logic of the algorithm for user grouping, rules for information recommendation, the list of expert-verified information, and the logic for user feedback evaluation can all be fine-tuned to reflect issues identified from the data analysis. In the *review and* *optimize* stage, the best configuration for the information recommendation logic should be determined based on testing different configurations. It is most practical to conduct tests on different parts of the information recommendation in parallel with tests in the operate stage of the software to minimize impact to the existing system. To achieve this, a staging infrastructure can be set up, where a mirror copy of the solution can be created for test-related activities.

### Implementation Considerations

#### Overview

This methodology can be adopted for the implementation of health information recommendation functionalities, either with already launched applications or when the application is still under development. In the next 2 subsections, we present implementation-related considerations of the methodology: first, components need to be developed and how these interoperate with other application components is described; and second, a 2-phased implementation strategy that aims to provide the optimal UX is described.

#### Software Components

**Figure 3:**
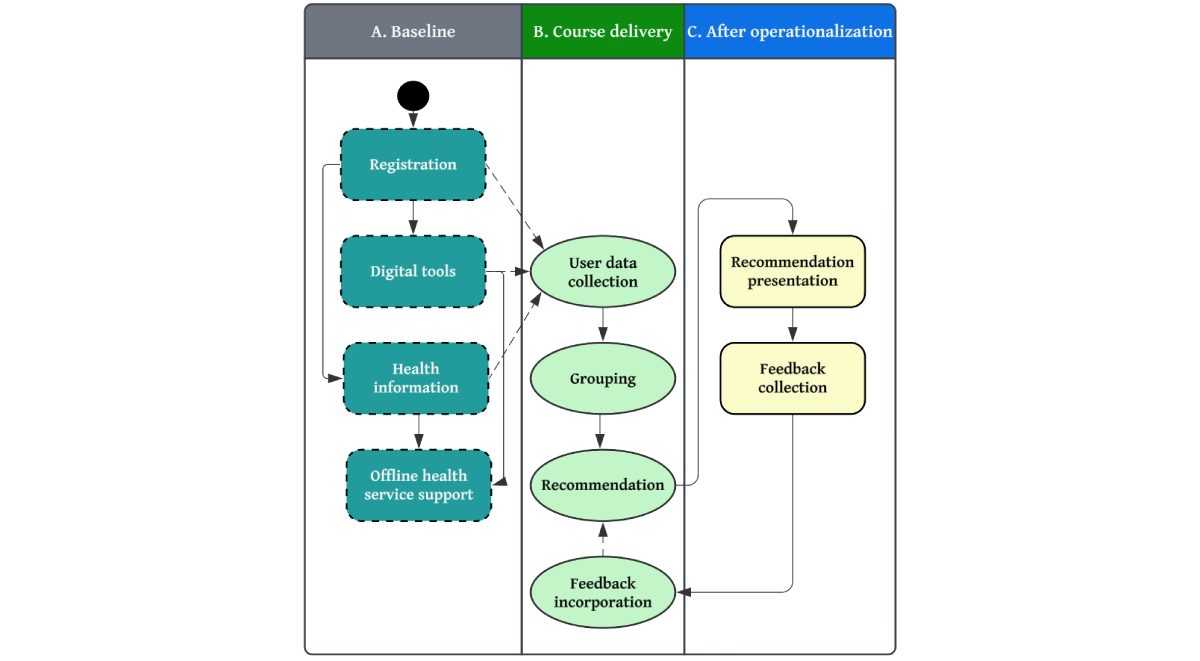
Software component design. (A) Components of a typical web-based health information application. (B) Backend components of the information recommendation solution. (C) Front-end components of the information recommendation solution.

The architecture design for the information recommendation solutions could vary vastly due to factors such as user requirements, the software technology stack being applied, the skill sets of developers, and governance restrictions (eg, the General Data Protection Regulation applicable in the European Union). However, when adopting the methodology, logical components for the health information recommendation functionality should be consistent. Figure 3 shows a high-level software component diagram that implements health information recommendation functionalities in a web-based health information application. The diagram consists of 3 sections: components of a typical web-based health information application (Figure 3A), backend components of the information recommendation solution (Figure 3B), and front-end components of the information recommendation solution (Figure 3C).

Figure 3A shows that a typical web-based health information application in the form of a website could include web pages of health information; instructions on local offline support; and, optionally, digital tools for certain informatic purposes and a registration component that is often needed by the digital tools. The 4 backend components shown in Figure 3B are needed for an information recommendation, which collects and processes all user input data, makes recommendations, and incorporates user feedback. Depending on the design of the information recommendation logic, the user input data collected could include user account data, health information browsing history, digital tool input, and user feedback on recommendations. The output of the 4 backend components includes updated information recommendation logic and a list of recommendations ranked based on user feedback. The front-end of the information recommendation solution shown in Figure 3C comprises 2 components: one for presenting recommendations and the other for collecting recommendation feedback. Depending on the design of the user engagement process, these 2 components can be either allocated on dedicated web pages or integrated into the web pages of any web-based health information application component.

The software components are designed in a loosely coupled fashion, where all functions and algorithms are independently maintained. Such a design pattern makes the adjustment of the information recommendation logic possible, from fine-tuning to a total replacement of the recommendation model. This feature is critical to a phased implementation of the solution, as will be discussed in the next subsection. One additional advantage of such a design is that it enables the potential of the web-based health information application to become a test bed of information recommendation algorithms, where algorithms can be easily alternated to test performance.

#### Implementation Phases

When the information recommendation solution is first launched, the number of users is small, and feedback on recommendations is not yet provided. Here, an information recommendation model that relies heavily on crowdsourced data for recommendation evaluation could produce suboptimal recommendations, impacting the UXs with the web-based health information application. To ensure the quality of recommendations before crowdsourced data are sufficiently accumulated, an implementation strategy is applied with the following two phases: (1) an initialization phase, in which crowdsourced data are not yet sufficiently collected, and an initial version of the algorithm for recommending information is used, where the information recommendation logic does not rank the recommendations based on user feedback data; and (2) an execution phase, where crowdsourced data are sufficient, and an execution version of the algorithm for recommending information is used, where user feedback is incorporated into the information recommendation logic for ranking recommendations based on user feedback data.

The main difference between these 2 versions of the algorithms is their logic in dealing with user feedback. Specifically, in the initial version of the algorithm, the list of recommendations is generated purely based on medical experts’ input (ie, a set of predefined rules for information recommendation), whereas in the execution version, the list of recommendations is generated based on medical experts’ input and further ranked based on user feedback data. Due to the loosely coupled software design, the algorithm for recommending information can be easily replaced. Researchers and data analysts can decide it is time to replace the algorithm when the amount of user feedback data is sufficient for the execution version of the algorithm to execute effectively.

### Ethical Considerations

The development of the platform and analysis of EndoZone data was approved by the University of Adelaide Human Research Ethics Committee (H-2020-013 & H-2023-054). Informed consent was obtained from community members participating in the design and development phase of the EndoZone informatics platform, and all users accessing the tool after it was launched online. The extraction and analysis of de-identified EndoZone platform data for this study was in accordance with the guidelines approved by the ethics committee.

## Results

### Case Study: EndoZone Informatics

The methodology for enabling health information recommendation functionalities has been successfully applied in the development of the information recommendation functionalities of a co-designed endometriosis information platform called EndoZone [14]. Endometriosis is a chronic condition, where tissue similar to the lining of the uterus develops in places outside the uterus. Symptoms of endometriosis may include pain with menstruation, chronic pelvic pain, fatigue, and subfertility. Globally, it is estimated that endometriosis affects approximately 190 million women and people presumed female at birth [32]. To address the wide-ranging impact of endometriosis, the Australian government and Jean Hailes for Women’s Health funded the development of EndoZone to improve knowledge, address symptoms, and provide strategies for managing endometriosis. This platform was designed for people affected by the condition as well as their supporters, such as parents, partners, teachers, and coworkers. The platform was cocreated and developed using the design thinking framework. During the cocreation process of the EndoZone platform, endometriosis community focus groups (n=36) were held to explore the key challenges and needs of the endometriosis community; in addition, a community priorities survey was conducted with 347 community member responses. On the basis of the key priorities identified, it was decided that functionalities would be developed to facilitate interaction and to support people experiencing endometriosis symptoms through the recommendation of strategies based on their symptoms, that is, EndoZone Informatics. The design, development, and implementation of EndoZone Informatics strictly follows the health information recommendation methodology. The solution was co-designed with other components of the EndoZone platform and integrated into the platform in April 2023. The solution is currently fully implemented and operating in the execution phase. In the following subsections, we present the design, development, and implementation of EndoZone Informatics to showcase the practicality of adopting the methodology for the design and implementation of information recommendation functionalities in a web-based health information application.

### Design and Develop

The design of EndoZone Informatics was part of the broader platform development process, which follows the broader co-design process of EndoZone. It was designed in consultation with 5 community representatives from endometriosis associations (patients, advocates, and supporters), clinicians (endometriosis or fertility specialist, physiotherapist or pain researcher, and endometriosis nurse), researchers, and 2 health informatics specialists. This involved a series of workshops and meetings to discuss details of the user engagement process as well as a smaller working group with clinicians to develop the initial information recommendation logic. The design was mocked up in consultation with the UI or UX designer and then integrated into the EndoZone platform. The outcome of this co-design process includes the user engagement process and the corresponding UI or UX prototype, a feedback collection method using email invitations and web-based surveys, and the information recommendation logic. Specifically, the information recommendation logic includes a list of 16 expert-verified articles for different endometriosis self-management therapies; a set of 27 rules that match symptoms to the recommendation of therapies (eg, one rule is that if the user experiences severe menstrual cramps, an article on transcutaneous electrical nerve stimulation therapy will be recommended); and algorithms for user grouping, analyzing feedback, feedback incorporation, and recommending information. After the design was ready to be reviewed, a review meeting was carried out for all stakeholders, where feedback on the design outcome was collected for adjustment. After the design outcome was adjusted and agreed upon, the solution designer and the UI or UX designer translated the outcomes to formal UI or UX design and architecture specifications for the development work to be carried out.

The develop, test, and deploy process was carried out using an agile approach, more specifically, the Scrum development process [33], which is preferred by the development team due to the existing software technology stack and developer skill sets. The development progress was regularly reported to, and closely monitored by, the digital health solution transformation experts. One issue that was encountered during this stage was some previously unforeseeable dependencies of the informatics components on several other components of the platform, which caused a 3-month delay in the release date of EndoZone Informatics. However, the development process is in general smooth.

To test EndoZone Informatics, medical and health experts participated in 2 demonstrations of the platform and tested the ready-to-launch version. In all, 9 community users participated in testing the platform’s early versions through a beta version with restricted access. Specifically, the user test was conducted after a series of tasks in which user testers were audio and video recorded while they completed the tasks and provided verbal feedback as they were using the platform. They also completed a series of questions related to their feedback on the platform (eg, what they liked, what they did not like, and suggestions for improvement), the usability of the platform, and whether they would recommend the platform to a friend or colleague. The feedback obtained from the user test was then incorporated into the further development of EndoZone Informatics features.

### Operate

**Figure 4:**
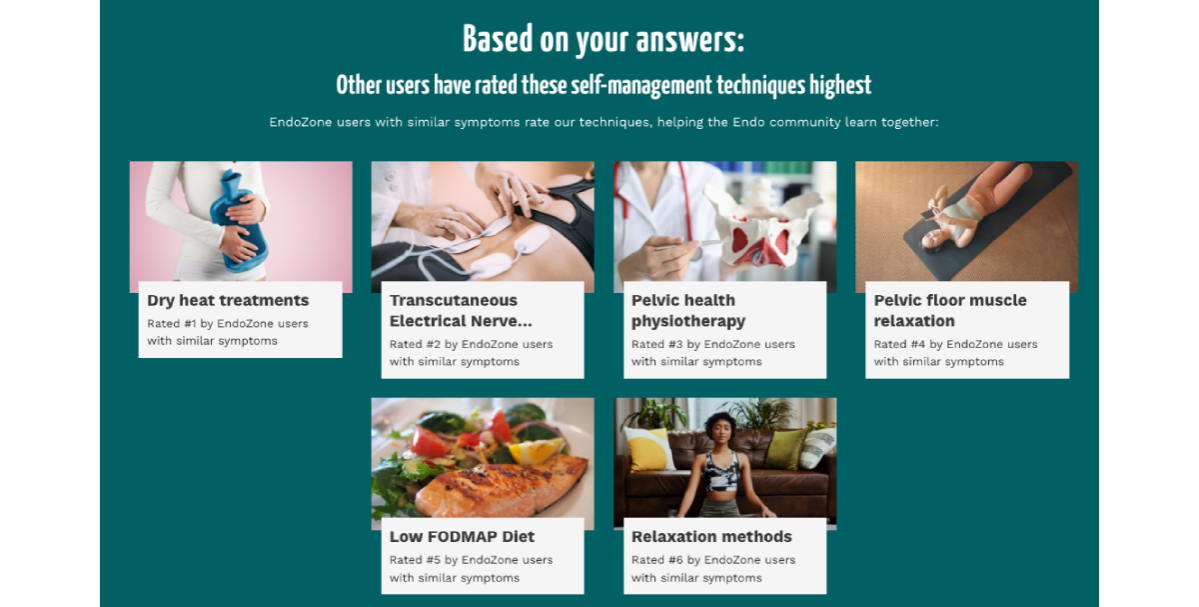
Recommended self-management therapies.

**Figure 5:**
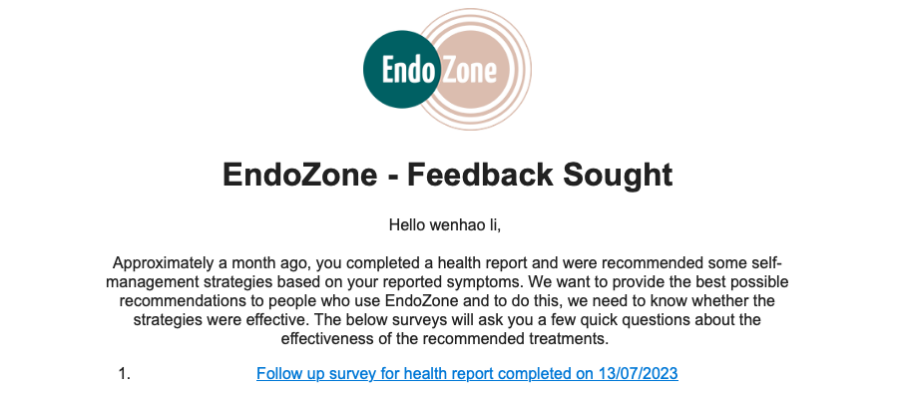
Invitation email to a follow-up survey.

**Figure 6:**
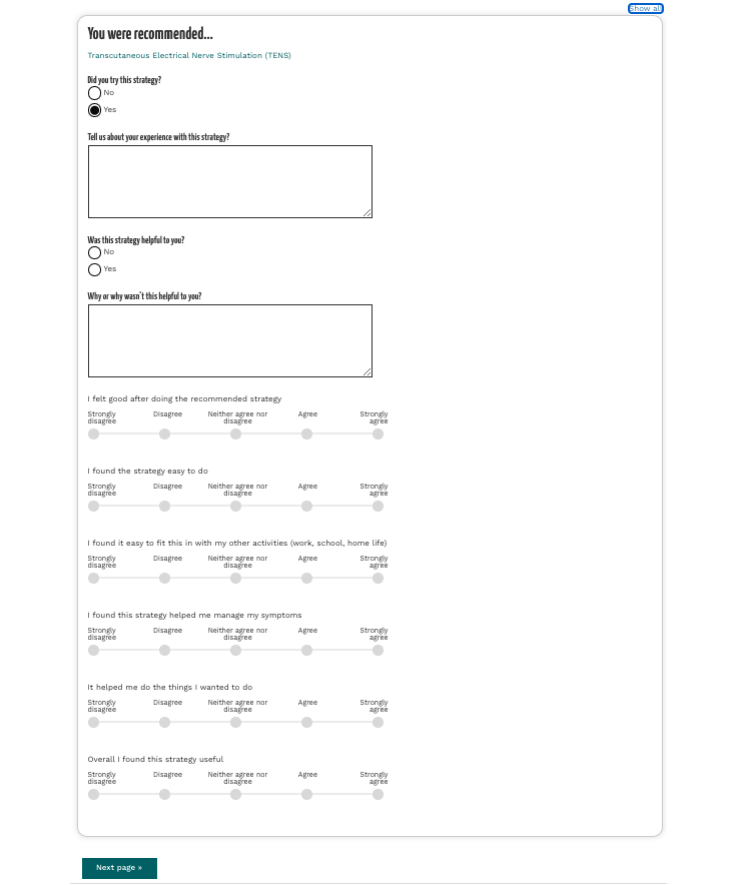
A follow-up survey example.

A user starts to engage with the EndoZone information recommendation solution from the submission of a health questionnaire named *My Endo Report*. The questionnaire contains a series of questions related to self-reported endometriosis symptoms and treatments that have been tried to manage symptoms, as well as a brief medical history. After the user has submitted the questionnaire, the backend algorithms are triggered to produce a list of recommended self-management therapies, where the recommendations are presented as part of *My Endo Report* (Figure 4). In EndoZone, the crowdsourced input (ie, user-provided feedback on recommended therapies) is used to determine the order of the recommendations being presented: among users with similar symptoms, therapies that are rated as “more useful” are given a higher rank and shown first in the list of recommendations. The description text of each recommendation contains a ranking to highlight this order. If the user clicks on a recommended therapy, the solution assumes that the user has viewed the content and records the click event. Next, 30 days after the recommendations are made, an email is sent to the user, inviting the user to complete a follow-up survey regarding the recommendations (Figure 5). When the user accepts the invitation, a follow-up survey is generated, containing questions related only to the recommendations that the user has clicked on. For each recommended self-management strategy, the survey contains 10 questions. It asks the user about the usefulness of the strategy, including their feelings after practicing the strategy, the practicality of the strategy, the effectiveness of the strategy in improving their symptoms, and so on. Figure 6 shows an example follow-up survey for the recommendation of pelvic health physiotherapy. Once the user has submitted the follow-up survey, their engagement with the EndoZone information recommendation solution is complete.

### Software Architecture

The architecture design in the EndoZone information recommendation solution strictly followed the component design shown in Figure 3 but was customized to fit the specific requirements of the application. First, based on the needs of the EndoZone information recommendation logic, the user data collection component only collects user registration data (ie, demographic profile data) and user input to the digital tool (ie, *My Endo Report* submission data). Second, the recommendation presentation component is integrated into the *My Endo Report* summary page of the application as part of the *My Endo Report* outcome.

Deployment wise, based on best practice, the EndoZone information recommendation solution is designed to be cloud based. It operates on cloud-based infrastructure using Amazon Web Services. All recommendation-related components are deployed in the form of microservices using Amazon Web Services Lambda, where each microservice contains components that are needed for a single application programming interface call. Specifically, the user data collection, grouping, and recommendation components are deployed in 1 microservice. Once the *My Endo Report* questionnaire is submitted, this microservice is called and responds with a list of recommended self-management therapies. The feedback incorporation component is deployed in another microservice. Once the follow-up survey is submitted, this microservice is called to update the information recommendation logic.

### Implementation

The implementation process of the EndoZone information recommendation solution followed the 2-phased process. Compared with what is described in the *Overview* subsection in the *Methods* section, an alternative data accumulation approach was conducted in the initialization phase to accelerate the transition to the execution phase. After the platform was launched, a targeted social media campaign on Instagram and Facebook was conducted to promote initial use of the platform. During the campaign, the initial version of the algorithm for recommending information was executed based on the expert-derived set of information recommendation rules that were matched to self-management therapies and symptoms that were indicated in *My Endo Report*. In completing the report, users are contacted via various channels to self-rate how helpful each self-management therapy or strategy was to manage their symptoms using a 3-point scale (“Didn’t work,” “Helped a bit,” and “Helped a lot”).

At the time of reviewing the data, the EndoZone platform had had 57,000 visitors (Google Analytics; February 20, 2024), predominantly from Australia (n=32,000, 56.14%), the United States (n=6000, 10.53%), the United Kingdom (n=5200, 9.12%), and New Zealand (n=5000, 8.77%), of whom 5756 (10.1%) completed *My Endo Report* and submitted it through the platform. User feedback data were aggregated to count the number of reports that indicated that a particular strategy either “Helped a bit” or “Helped a lot.” This feedback was then considered to be the initial rating of therapies on which the execution version of the algorithm could rely; for example, *yoga* was rated by 682 people, of whom 404 (59.24%) rated it as either “Helped a bit” or “Helped a lot.” These feedback data were then manually incorporated, where a rating of “404/682 (59.24%)” was set as the initial rating of the therapy *yoga* for all user groups. A further analysis of the data collected through the platform is being conducted to feed into the next iteration of EndoZone Informatics.

### Review and Optimize

The EndoZone information recommendation solution was integrated into the EndoZone platform in April 2023. Tests and feedback from the volunteer group have shown that the overall user engagement process can be carried out well, with a good UX. Meanwhile, based on early data accumulated, several design issues have been identified; for example, the participation rate for providing feedback is lower than expected. We suspect that the UI or UX design could be the major cause for this outcome: first, in the current design, only registered users are invited to complete the follow-up survey (unregistered users cannot be invited because they are not asked for their email address). Currently, most users use the site anonymously, which means that most users of the platform who decided not to create an account in EndoZone are not able to experience the full recommendation functionalities and provide recommendation feedback. Second, the recommendation section is in a relatively inconspicuous position on the *My Endo Report* summary page. This may lead to reduced visibility and hence less user participation. The finding indicates that the design of web pages (UI or UX) is highly relevant to the effectiveness of the solution. It also indicates that the methodology is limited in identifying specific design defects during the initial *design and develop* stage. However, such defects can be addressed in the *review and* *optimize* stage, where issues that crop up during the execution of the solution are reviewed. In the context of the EndoZone platform, remedial development work has been planned in the second phase of the project from 2024 to 2026.

Furthermore, the logic for tracking user engagement with recommended therapies (ie, once the article is opened, the recommended therapy is considered to have been read) is not consistent with the industrial standard that large IT companies have applied; for example, in Google Analytics, a user is considered to have engaged with a web page if they stay on the page for >10 seconds [34]. How user engagement is tracked is not defined by the methodology and could vary from case to case. However, in the context of the EndoZone platform, the solution logic does not cause a loss of user feedback data. The impact on the UX (ie, several more survey questions are asked regarding a therapy that the user has not practiced) is limited and can be eliminated by adjusting the questions in the follow-up survey.

## Discussion

### Outcome

In the previous sections, we have presented a methodology that enables health information recommendation functionalities in web-based health information applications. The concept of the methodology as well as the implementation considerations, including the software component design and the 2-phased implementation process, are described in detail, based on which information recommendation solutions can be created and operationalized. The methodology has been refined and validated through its application to create EndoZone Informatics, that is, the information recommendation solution of an endometriosis information platform named EndoZone. Early data from the execution of the EndoZone Informatics solution shows that using this methodology was effective in recommending medical expert–verified information while incorporating crowdsourced input from users with similar conditions. This methodology helped users to find the information that could be of most use to them. The loosely coupled software component design enabled high flexibility in adjusting the information recommendation model, which makes the 2-phased implementation process easy to carry out.

During the application of the methodology for EndoZone Informatics, we encountered several issues. To recap, first, the dependencies of the information recommendation components on other components of the web-based health information application caused a 3-month delay in the development progress of EndoZone Informatics. Second, the UI or UX design flaws, such as unregistered users not being able to experience the full recommendation functionalities and *underexposure* of the recommendation section in the *My Endo Report* summary page, have resulted in a lower-than-expected participation rate for providing feedback. These issues reveal a limitation of the methodology, that is, it is not able to address some specific software engineering problems. These issues also show the significance of the *review and optimize* stage, where design and development issues can be identified, and repair plans can be created.

In general, the application of the methodology for designing and implementing EndoZone Informatics is successful. It is a solid step toward enabling personalized information recommendation at scale. The solution indicates a promising approach where personalized health information recommendation can be enabled in all web-based health information applications. Compared with accessing health information via recommendations derived from commercial algorithms of search engines and social media platforms, a health information–access approach provides people with an alternative health information–ranking and –recommendation path, which ranks information based on people’s medical needs; provides them with trustworthy, credible, and evidence-based recommendations; and aims for the best health outcomes.

### Potential of the Methodology

The methodology is proposed to be applied in web-based health information applications targeting personalized health information recommendations for educational and knowledge-sharing purposes. As showcased by the EndoZone platform, this methodology is applicable and works well for web-based health information applications that share health information such as chronic disease self-management strategies. However, the practicality of applying the methodology in creating solutions for applications that target acute diseases is yet to be proven. Another area for further research is the practicality of applying this methodology for recommending clinical treatments. This requires systematic study of what the impact could be if the methodology was applied for recommending clinical treatments (eg, medication use). What kind of care decisions (safety or risk of harm or relative benefits) need to be considered? What are the ethical issues involved? Answers to these questions are not yet clear.

A promising area for applying the methodology concerns creating solutions for recommending other medical and health services, such as links to local medical experts, health services, advocacy organizations, and related web-based applications [35,36]. Exploring how solutions created by applying this methodology could help in connecting web-based services to local offline services to improve the quality and scope of user support would also be of value.

Another potential application of this methodology is to generate test beds for information recommendation algorithms and their suitability for different medical scenarios. As described in the *Overview* subsection of the *Methods* section, the software component design allows all key logic components to be independently maintained and easily replaced. This feature can be leveraged for new information recommendation algorithms or models to be tested; for example, by applying different information recommendation models and monitoring user interactions under each model, the performance of different information recommendation models can be analyzed.

### Conclusions

This study introduces a novel methodology that enriches web-based health applications with personalized information recommendation capabilities. Tested through the development of the EndoZone platform, our approach successfully merges expert knowledge with user insights to provide targeted health information. While we encountered developmental and design challenges, these experiences highlighted the importance of adaptability and continuous refinement. The methodology’s potential extends beyond the specific case of EndoZone, offering a scalable solution for tailoring health information across various authoritative health websites, with implications for improving patient education and engagement in a digital era.

## Abbreviations

UI: user interface
UX: user experience
